# Human leukocyte antigen I is significantly downregulated in patients with myxoid liposarcomas

**DOI:** 10.1007/s00262-021-02928-1

**Published:** 2021-04-24

**Authors:** Naoki Oike, Hiroyuki Kawashima, Akira Ogose, Hiroshi Hatano, Takashi Ariizumi, Tetsuro Yamagishi, Yudai Murayama, Hajime Umezu, Chihaya Imai, Masanori Hayashi, Naoto Endo

**Affiliations:** 1 Division of Orthopedic Surgery, Graduate School of Medical and Dental Sciences, Niigata University, 1-757 Asahimachi-dori, Niigata, 951-8510 Japan; 2 Department of Pediatrics, University of Colorado, Aurora, CO USA; 3 Department of Orthopedic Surgery, Uonuma Kikan Hospital, Niigata, Japan; 4 Department of Orthopedic Surgery, Niigata Cancer Hospital, Niigata, Japan; 5 Department of Pathology, Graduate School of Medical and Dental Sciences, Niigata University, Niigata, Japan; 6 Department of Pediatrics, Graduate School of Medical and Dental Sciences, Niigata University, Niigata, Japan

**Keywords:** CD163+ macrophages, Human leukocyte antigen I, Prognosis, Programmed death ligand 1, Liposarcomas, Tumor immune microenvironment

## Abstract

The characteristics of the tumor immune microenvironment remains unclear in liposarcomas, and here we aimed to determine the prognostic impact of the tumor immune microenvironment across separate liposarcomas subtypes. A total of 70 liposarcoma patients with three subtypes: myxoid liposarcoma (*n* = 45), dedifferentiated liposarcoma (*n* = 17), and pleomorphic liposarcoma (*n* = 8) were enrolled. The presence of tumor infiltrating lymphocytes (CD4+ , CD8+ , FOXP3+ lymphocytes) and CD163+ macrophages and expression of HLA class I and PD-L1 were assessed by immunohistochemistry in the diagnostic samples; overall survival and progression-free survival were estimated from outcome data. For infiltrating lymphocytes and macrophages, dedifferentiated liposarcoma and pleomorphic liposarcoma patients had a significantly higher number than myxoid liposarcoma patients. While myxoid liposarcoma patients with a high number of macrophages were associated with worse overall and progression-free survival, dedifferentiated liposarcoma patients with high macrophage numbers showed a trend toward favorable prognosis. Expression of HLA class I was negative in 35 of 45 (77.8%) myxoid liposarcoma tumors, whereas all dedifferentiated liposarcoma and pleomorphic liposarcoma tumors expressed HLA class I. The subset of myxoid liposarcoma patients with high HLA class I expression had significantly poor overall and progression-free survival, while dedifferentiated liposarcoma patients with high HLA class I expression tended to have favorable outcomes. Only four of 17 (23.5%) dedifferentiated liposarcomas, two of eight (25%) pleomorphic liposarcomas, and no myxoid liposarcoma tumors expressed PD-L1. Our results demonstrate the unique immune microenvironment of myxoid liposarcomas compared to other subtypes of liposarcomas, suggesting that the approach for immunotherapy in liposarcomas should be based on subtype.

**Supplementary Information:**

The online version contains supplementary material available at 10.1007/s00262-021-02928-1.

## Introduction

Liposarcomas (LPS) are malignant tumors of adipocytic differentiation and the most common soft tissue sarcoma subtypes, comprising approximately 15%–20% of soft tissue sarcomas in adults [1, 2]. The 2020 World Health Organization classification [3] lists five histological subtypes for LPS: the intermediate atypical lipomatous tumor/well-differentiated liposarcoma (WDLPS), the malignant dedifferentiated liposarcoma (DDLPS), myxoid liposarcoma (MLPS), pleomorphic liposarcoma (PLPS), and myxoid pleomorphic liposarcoma. Each subtype has their own distinct clinical features. WDLPS and DDLPS represent the most common type of LPS, accounting for approximately 40–45% of LPS [3]. Both WDLPS and DDLPS usually exhibit a supernumerary ring and/or a giant rod chromosome with the amplification of 12q13-15, which contains multiple genes that have been indicated to be contributing to the oncogenesis, such as MDM2, CDK4, HMG2A, and YEATS4 [2]. While the overall mutational burden of WDLPS is low, it is believed that the accumulation of additional genetic mutations leads to the development of DDLPS [1, 2].

MLPS is the second most common type of liposarcomas, comprising 20–30% of liposarcomas [3]. Molecularly, it is characterized by a chromosomal translocation t (12;16)(q13; p11) that results in the FUS-DDIT3 (or CHOP) fusion protein in over 90% of patients, with a small number with a EWSR1-DDIT3 translocation [2, 3]. Distant metastasis can commonly arise in various sites such as bone, retroperitoneum, and serosal surfaces, even in the absence of lung metastasis [2, 3].

PLPS is the rarest variant of LPS, accounting for 5% of all LPS [3]. PLPS is also the most aggressive LPS subtype with a high rate of recurrence and metastasis [2]. However, current understanding of the molecular pathology of PLPS is limited by the rarity of this disease [1]. PLPS tends to show a complex karyotype including multiple chromosomal losses and gains, indicating a pathogenesis driven by complex and variable genomic aberrations [2].

Currently, wide-margin surgical resection remains the core curative option for LPS [2, 3], and perioperative radiation is often offered to reduce local recurrence [4, 5]. However, distant metastasis is not uncommon, and prognosis is exceptionally poor for these patients, with the use of chemotherapy and radiotherapy limited to advanced or recurrent cases [2]. The limitation in current treatment for aggressive LPS emphasizes the need for effective new systemic therapeutic approaches, such as immunotherapies.

Interaction between programmed cell death 1 and programmed death ligand 1 (PD-L1) plays an important role in tumor evasion through T cell inactivation. Previous research has demonstrated that high expression of PD-L1 correlates with worse prognosis in several malignancies [6, 7]. While there have been reports indicating PD-L1 expression as a poor prognostic indicator in soft tissue sarcomas, these studies consisted of only a small number of LPS patients with all the subtypes lumped together. With the more current understanding of the molecular heterogeneity, further investigation of PD-L1 expression and the immune landscape in each subtype of LPS is warranted [8, 9].

HLA class I proteins are expressed on virtually all nucleated cells and have several important functions in adaptive immunity [10]. HLA class I proteins can present foreign antigens to cytotoxic T cells either on antigen presenting cells such as dendritic cells or target cells, a process that is highly regulated. Furthermore, HLA class I proteins function as one of the most important inhibitory signals for natural killer (NK) cells, aiding NK cells to recognize non-self-cells by the lack of HLA class I proteins [10]. NK cells are a critical effector of antitumor innate immunity in cancer immune surveillance, and adoptive transfer of NK cells is considered an attractive immunotherapeutic option in patients with hematological malignancies and solid tumors [10, 11].

The characteristics of the tumor immune microenvironment in each LPS subtype has not been assessed in a systemic fashion with survival outcome available. The aim of the current study is to assess the tumor immune microenvironment according to the distinct subtypes of LPS, ultimately to aid in the design of effective immunotherapeutic approaches in patients with LPS according to the distinct subtypes.

## Materials and methods

### Patients and samples

Primary LPS patients with dedifferentiated liposarcoma (DDLPS), myxoid liposarcoma (MLPS), and pleomorphic liposarcoma (PLPS), who were diagnosed and treated at the University of Niigata between 1991 and 2018, were enrolled in this retrospective study. Patients with well-differentiated liposarcomas were excluded. Patients who did not have samples available prior to systemic therapy were excluded. A total of 70 patients were identified, and diagnostic samples (or surgical samples if upfront surgery was performed) were used for immunohistochemical examination. All samples were obtained by an open biopsy, with the exception of one sample obtained by a core needle biopsy. Among these patients, 16 patients also had metastatic tumor samples available. In all cases, hematoxylin and eosin staining slides were reviewed to confirm that the blocks included adequate viable tumor cells. Molecular diagnostic testing results, such as FUS/EWS-DDIT3 fusion gene or amplification of MDM2, were collected when available. Clinical data were also extracted from medical records for statistical analysis.

This study was approved by the Institutional Review Board of Niigata University (No. 2016–0024) and was conducted in accordance with the Declaration of Helsinki. All patients gave written informed consent prior to participation in this research.

### Immunohistochemistry

Immunohistochemical staining for tumor infiltrating lymphocytes (TILs; CD4, CD8, FOXP3), CD163+ macrophages, HLA class I, and PD-L1 were carried out as previously described [12]. Slides were stained with the primary antibodies summarized in Supp. Table 1. Next, the slides were treated with Histofine Simple Stain MAX PO MULTI (Nichirei Bioscience, Tokyo Japan), and the peroxidase activity was detected with Simple Stain DAB (Nichirei Biosciences). Finally, slides were counterstained with hematoxylin (Vector Laboratories Inc., Burlingame, CA). Appropriate positive and negative control was prepared for CD4, CD8, FOXP3, CD163, and PD-L1. Intact expression of HLA class I was confirmed by staining of endothelial cells.

### Evaluation of immunohistochemistry

To enumerate tumor infiltrating lymphocytes and macrophages, areas with the most abundant lymphocytes or macrophages were selected in each section at a low power magnification. The sections were then photographed with an Olympus DP73 digital camera (Olympus, Tokyo, Japan) from maximum of five high power fields (×200), and cells were counted manually. The count was conducted two times by an experienced pathologist who was blinded to the clinical information of the patients, and the average number was used as the final value of lymphocytes and macrophages in each patient. Then, the median value was used to distinguish the patients into a high or low infiltration group. For HLA class I, we graded the expression status according to previous reports [12]: high (number of positive cells ≥ 50%), low (≤ 5% number of positive cells < 50%), and negative (number of positive cells < 5%). PD-L1 positivity was defined when the positive cells were more than 1% as previously reported [12].

### Statistical analysis

Statistical analyses were conducted with GraphPad Prism v8.0 software (La Jolla, California, USA). ANOVA test was used to evaluate the statistical significance between more than two groups. The Kaplan–Meier method was used to estimate overall survival (OS) and progression-free survival (PFS) probabilities in patients with MLPS and DDLPS. OS and PFS were measured from the date of the initial biopsy. A terminal point of OS was determined as the time to death or the time the patient was last seen. A terminal point of PFS was determined as the time of local recurrence, distant metastasis, disease progression, or last seen. Survival differences were analyzed by the log-rank test. A *p* value less than 0.05 was considered statistically significant.

## Results

### Clinical characteristics

Patient clinical characteristics are summarized in Table 1. There were 43 male patients and 27 female patients with a mean age of 57.7 years (18—86). Lung metastasis was observed in 3 of 70 patients at diagnosis. Six tumors were in the upper extremities, 59 tumors in lower extremities, and five tumors in the trunk. The disease stage was classified according to the American Joint Committee on Cancer 8th edition staging system [13], and 3 patients were stage II, 34 were stage IIIA, 30 were IIIB, and 3 were stage IV.

**Table 1:** Patient demographics and clinicopathological characteristics

Characteristic	Number of patients (%)
Total number of patients	70 (100)
*Histological subtypes*	
Dedifferentiated liposarcoma	17 (24.3)
Myxoid liposarcoma	45 (64.3)
Pleomorphic liposarcoma	8 (11.4)
*Gender*	
Male	43 (61.4)
Female	27 (38.6
*Location*	
Upper extremities	6 (8.4)
Lower extremities	59 (84.3)
Trunk	5 (7.1)
*Fusion gene status*	
Myxoid liposarcoma	
FUS-DDIT3	16 (35.6)
EWS-DDIT3	2 (4.4)
Unknown	27 (60)
*MDM2 amplification*	
Dedifferentiated liposarcoma	
Detected	10 (58.8)
Unknown	7 (41.2)
Pleomorphic liposarcoma	
Absent	5 (62.5)
Unknown	3 (37.5)
*Stage* (*AJCC system*)	
II	3 (4.3)
IIIA	34 (48.6)
IIIB	30 (42.9)
IV	3 (4.3)

*AJCC* American Joint Committee on Cancer system 8th edition

### Infiltration of lymphocytes and macrophages

**Fig. 1:**
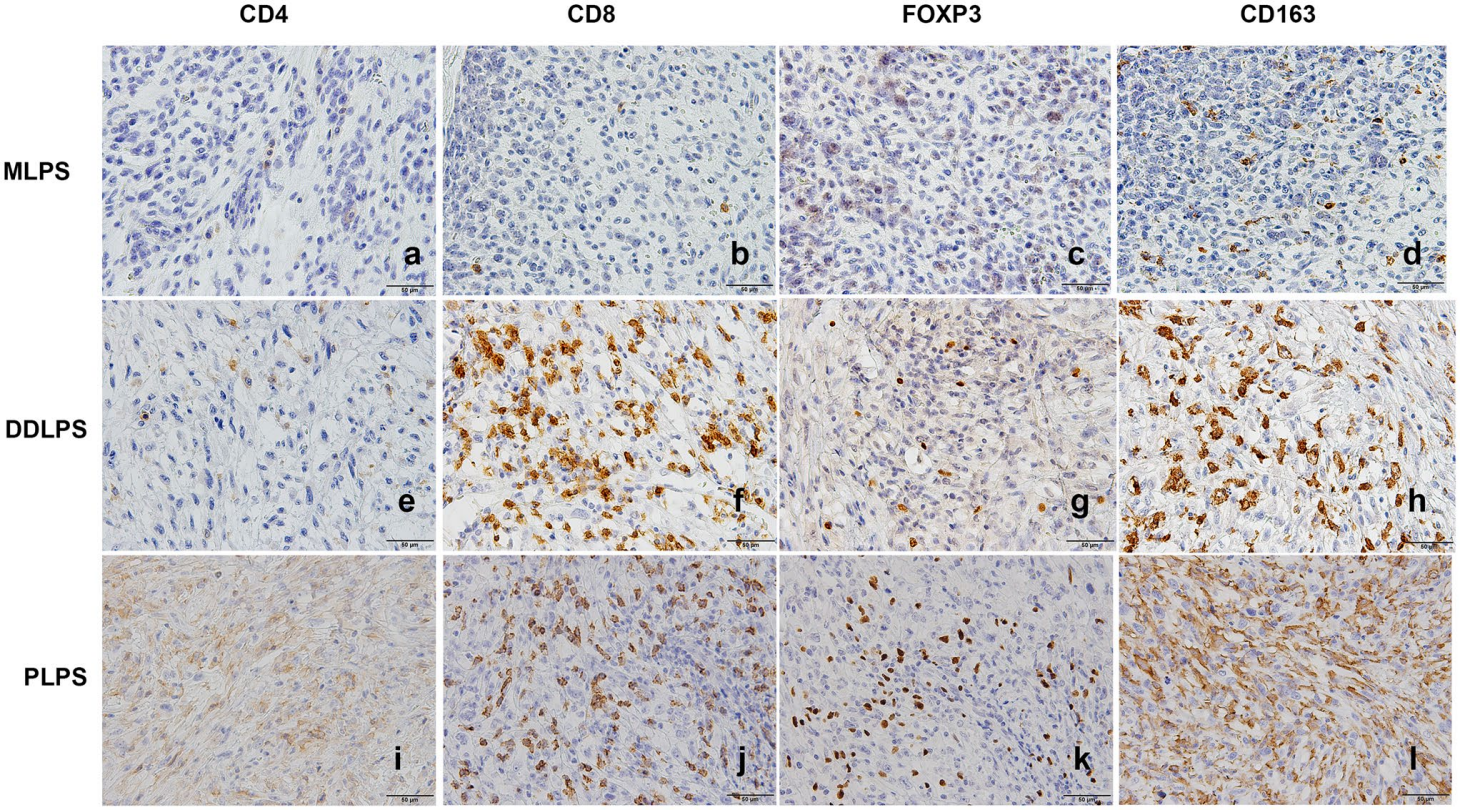
Tumor infiltrating lymphocytes (CD4+ or CD8+), regulatory T cells (FOXP3+), and macrophages (CD163+) were enumerated in each subtype of liposarcoma (200X). Scale bar represents 50 µm. **a–d** A MLPS sample with low to none CD4+ , CD8+ , FOXP3 lymphocytes, and low number of CD163+ macrophages. **e–k** CD4+ , CD8+ , FOXP3+ , and CD163+ macrophages were present in most samples of DDLPS and PLPS patients. *MLPS* myxoid liposarcoma, *DDLPS* dedifferentiated liposarcoma, *PLPS* pleomorphic liposarcoma

**Fig. 2:**
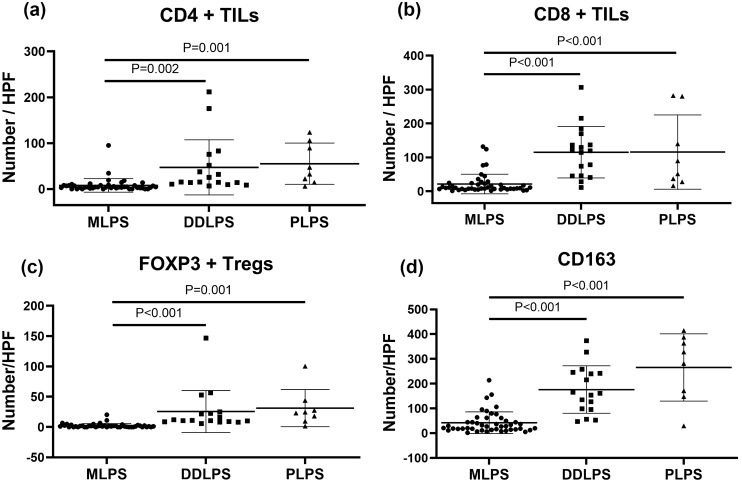
Number of CD4+ TILs, CD8+ TILs, FOXP3+ Tregs, and CD163+ Macrophages infiltrated into tumor microenvironment in each histological subtype. Number of CD4+ TILs (**a**), CD8+ TILs (**b**), and FOXP3+ TILs (**c**) and CD163+ macrophages (**d**) are significantly higher in DDLPS and PLPS than that of MLPS. Error bars show standard deviations. Tregs, Regulatory T cells. *TIL* tumor infiltrating lymphocytes, *MLPS* myxoid liposarcoma, *DDLPS* dedifferentiated liposarcoma, *PLPS* pleomorphic liposarcoma

TILs and macrophages were enumerated by averaging two separate counts from five high power fields per slide (Fig. 1a–k). TILs were defined by CD4 or CD8 positivity, and FOXP3 was used to further define CD4+ regulatory T cells. Macrophages were defined by CD163 positivity. The median numbers of TILs and CD163+ macrophages enumerated in each histological subtype were summarized (Table 2). Interestingly, samples from DDLPS patients and PLPS patients had significantly more TILs and macrophages identified than patients with MLPS (Fig. 2 a–d). Furthermore, 9% (4 of 45 samples) of MLPS patients had no CD4+ TILs, and 33% (15 of 45 samples) had no FOXP3+ Tregs. In contrast, all subtypes of TILs and macrophages were identified in all samples from patients with DDLPS and PLPS, highlighting the striking difference of immune infiltration among LPS subtypes.

**Table 2:** Number of infiltrated lymphocytes and macrophages

	CD4	CD8	FOXP3	CD163
MLPS	4.4 (0–95.2)	10.0 (1–131.8)	1.0 (0–20.2)	27.3 (1.6–213.8)
DDLPS	15.8 (6.6–212)	119.2(10.8–306.6)	11.0 (5.6–146.6)	161.6 (47–373.6)
PLPS	40.1 (6–123.6)	70.4 (16.6–282.2)	23.9 (1.4–100.4)	304.7 (29.4–414.2)

*MLPS* myxoid liposarcoma, *DDLPS* dedifferentiated liposarcoma, *PLPS* pleomorphic liposarcoma

### Expression of HLA class I and PD-L1

**Fig. 3:**
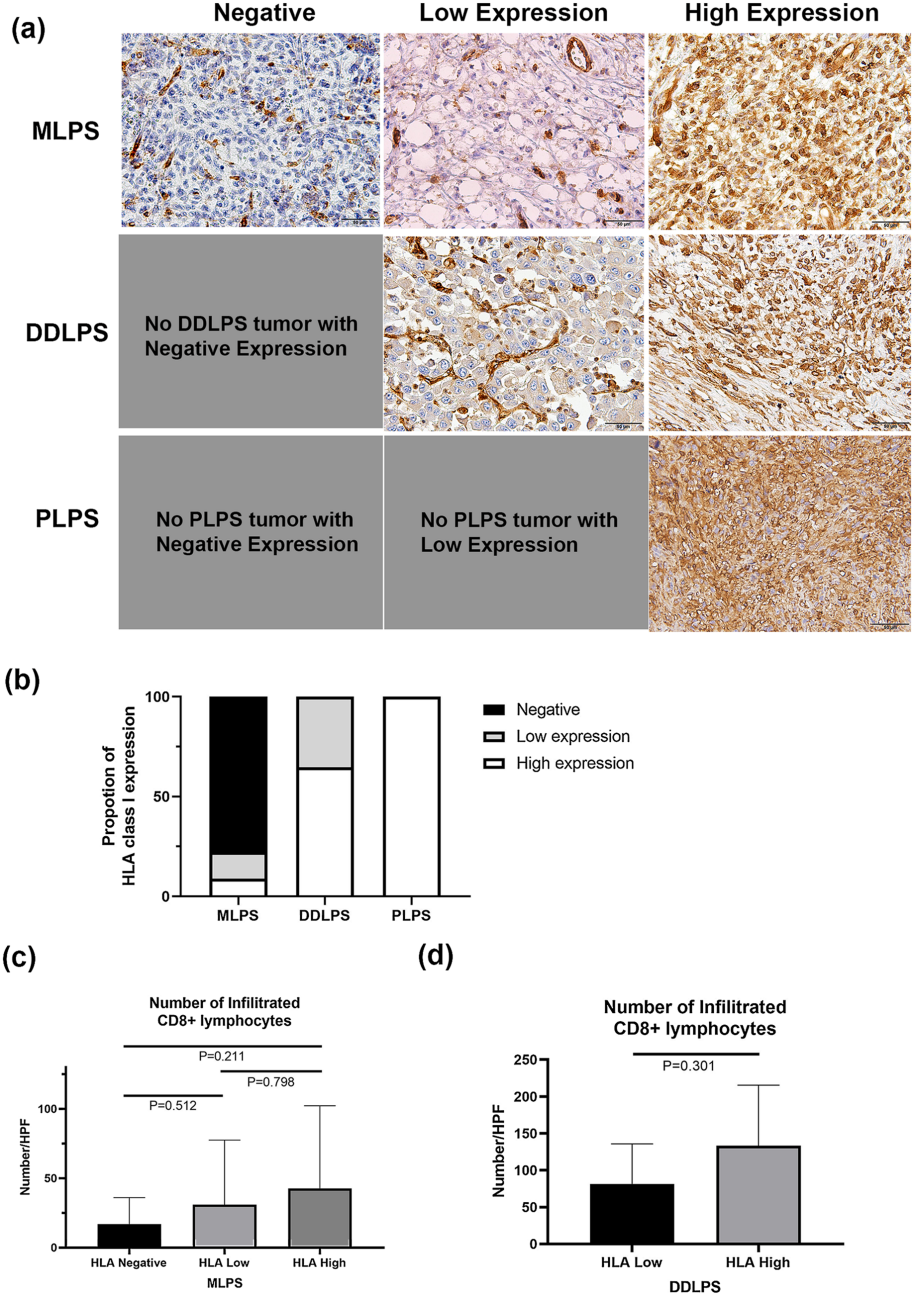
Expression of HLA class I in each histological type of Liposarcomas. **a** Various expression levels were found in patients with MLPS, whereas no patents were negative for HLA class I expression. Scale bar represents 50 µm, **b** most patients with MLPS showed lost or downregulation of HLA class I expression, **c** and **d** number of infiltrated CD8+ lymphocytes tend to be higher in patients with high expression of HLA class I in both MLPS and DDLPS. *MLPS* myxoid liposarcoma, *DDLPS* dedifferentiated liposarcoma, *PLPS* pleomorphic liposarcoma

Here, MLPS patients were found to have significantly less expression of HLA class I compared to DDLPS patients or PLPS patients (Fig. 3a). HLA class I was negative in 77.8% (35 of 45 samples), 13.3% (6 of 45 samples) had low expression, and only 8.9% (4 of 45 samples) had high expression in MLPS patients, whereas 64.7% (11 of 17 samples) of DDLPS patients and all 9 PLPS patients had high HLA class I expression (Fig. 3b). None of the DDLPS or PLPS patients had negative HLA class I expression. Next, we analyzed the relationship between HLA class I expression and infiltration of CD8+ lymphocytes in patients with MLPS and DDLPS. In both subtypes, patients with higher expression of HLA class I demonstrated a trend toward higher infiltration of CD8+ lymphocytes but did not achieve a statistical significance (Fig. 3c, d).

**Fig. 4:**
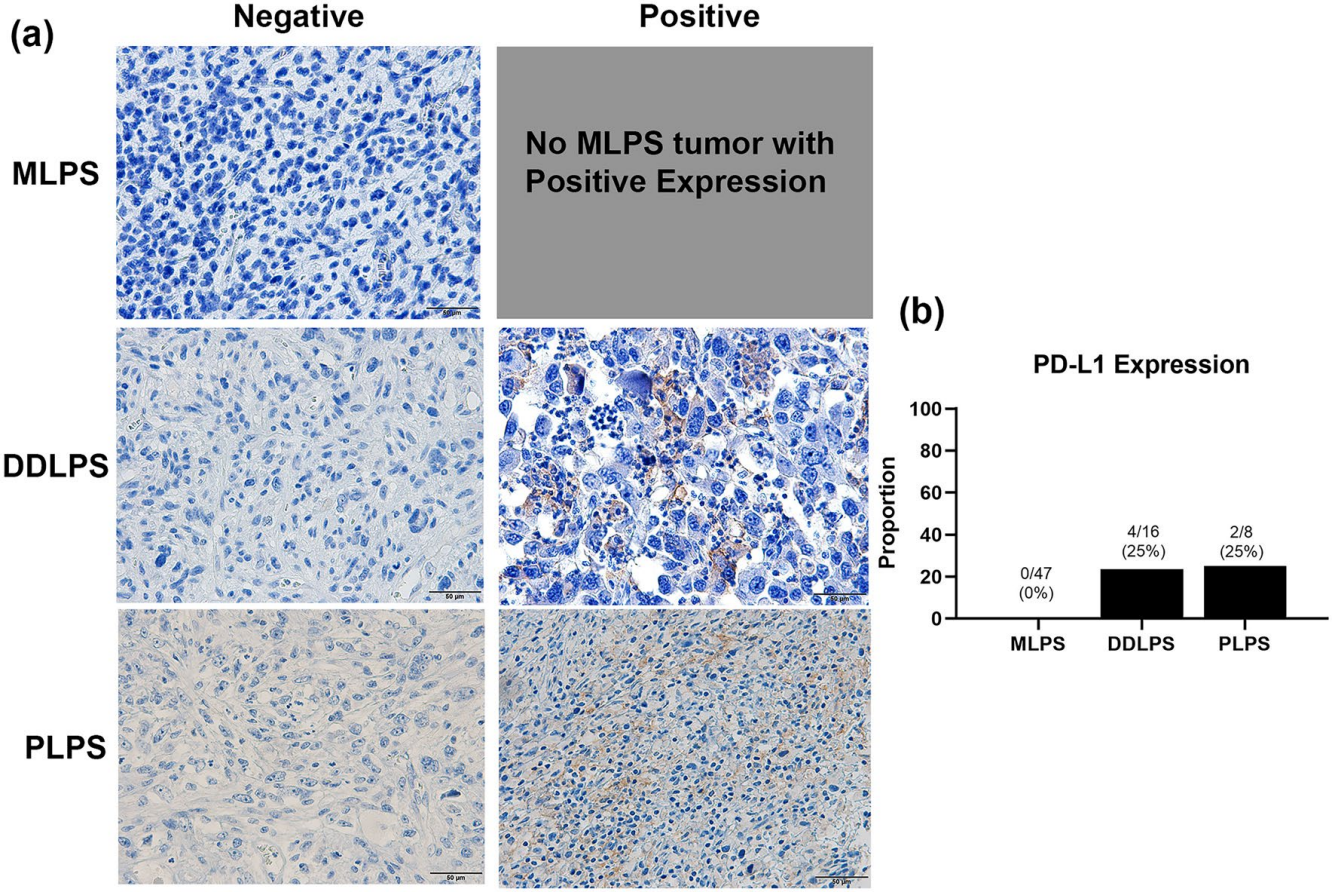
Expression of programmed death ligand 1(PD-L1) in each histological type of liposarcomas. No patients with MLPS were positive for PD-L1, while. 23.5% of patients with DDLPS and 25% of PLPS patients expressed PD-L1(**a**, **b**). Scale bar represents 50 µm. *MLPS* myxoid liposarcoma, *DDLPS* dedifferentiated liposarcoma, *PLPS* pleomorphic liposarcoma

For PD-L1, none of the MLPS patient samples expressed PD-L1, whereas 23.5% (4 of 17 cases) in DDLPS and 25% (2 of 8 cases) in PLPS were positive for PD-L1 (Fig. 4a, b). For the 16 MLPS patients where metastatic samples were available, HLA class I and PD-L1expression were evaluated, and all samples were negative for both HLA class I and PD-L1 (Supp. Fig. 1).

### Prognostic significance of the immune microenvironment

Next, based on IHC results, we investigated whether the tumor immune microenvironment at diagnosis correlated with patient outcome. The survival outcome data were available on all 70 patients with a follow-up range of 5–244 months. OS probabilities of patients with MLPS and DDLPS at 5 years were 72.2% and 50.0%, respectively. PFS probabilities of patients with MLPS and DDLPS at 5 years were 54.1% and 27.3%, respectively.

**Fig. 5:**
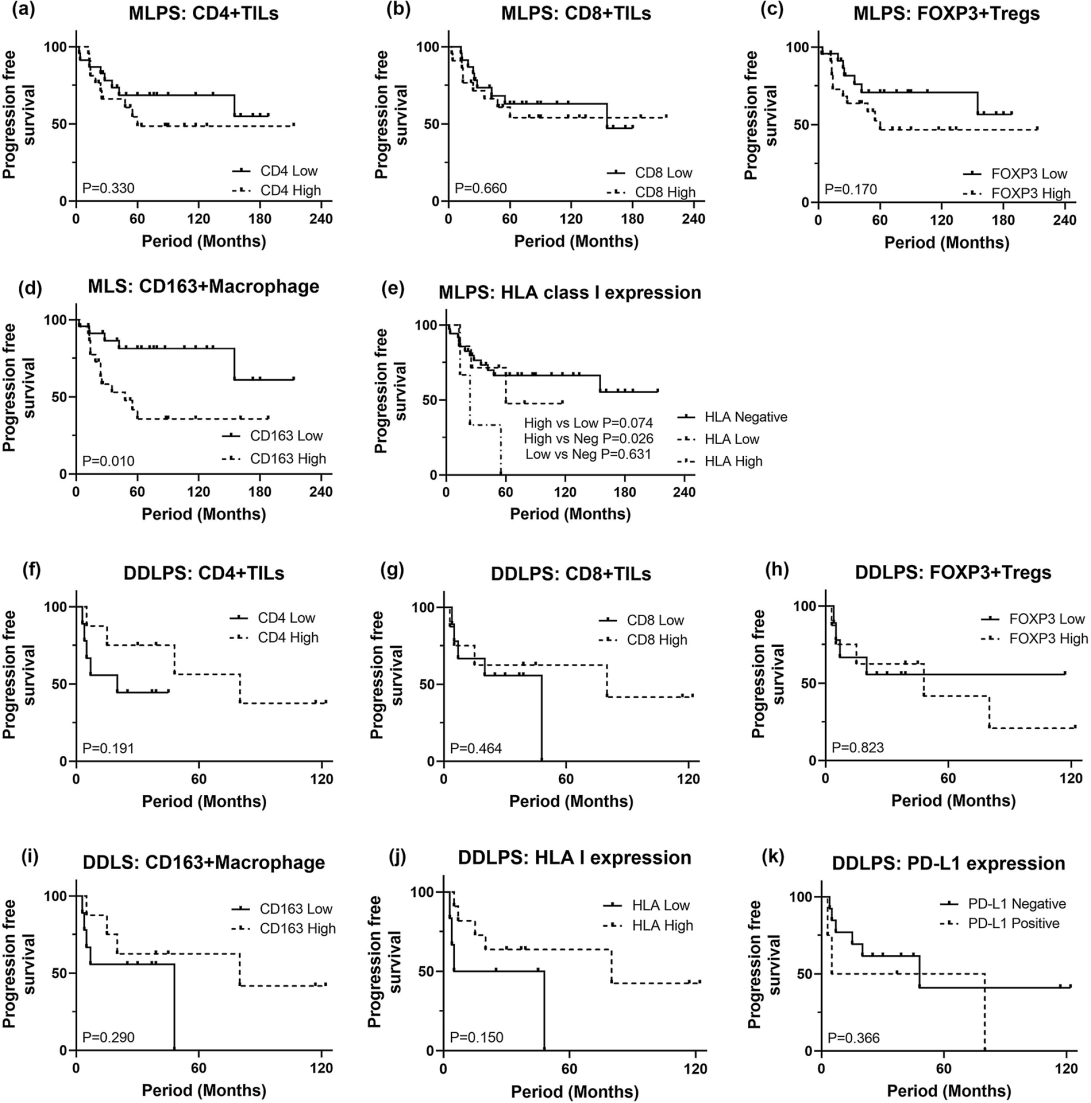
Kaplan–Meier curves illustrating progression free survival (PFS) in MLPS and DDLPS. Number of CD4+ TILs, CD8+ TILs, or FOXP3+ Tregs did not show significant impact on PFS (**a**–**c**), while higher number of CD163+ macrophages or high expression of HLA class I were associated with unfavorable PFS in patients with MLPS (**d**). No immune characteristics were found to be significantly associated with difference of PFS in patients with DDLPS (**f**–**k**). *MLPS* myxoid liposarcoma, *DDLPS* dedifferentiated liposarcoma

In MLPS patients, high number of CD163+ macrophages was significantly associated with unfavorable PFS (Fig. 5d) and OS (Supp. Fig. 2d). In patients with DDLPS, patients with higher number of infiltrating CD163+ macrophages demonstrated a tendency to have a favorable OS (Supp. Fig. 2i), although this did not reach statistical significance. CD4+ or CD8+ TILs or FOXP3+ Treg numbers did not demonstrate discernable survival tendencies (Fig. 5a–c, f–h, Supp. Fig. 2a–c, f–h).

For PD-L1 expression, there was no prognostic impact on OS or PFS in DDLPS patients (Fig. 5k, Supp. Fig. 2k). High expression of HLA class I showed unfavorable prognosis in patients with MPLS (Fig. 5e, Supp. Fig. 2e), and while not statistically significant, high expression of HLA class I tended to predict a favorable PFS in DDLPS patients (Fig. 5j).

## Discussion

While there have been some reports of investigating the tumor immune microenvironment of soft tissue sarcomas [8, 14, 15], there have been limitations, such as including a mixture of treated and untreated samples [14], or lumping all the subtypes of LPS together, despite the known differences in biology and clinical behavior among the subtypes of LPS [8, 15]. This study is the first large study to systematically characterize the tumor immune microenvironment and correlative outcome in patients with LPS based on histological subtype.

The mechanism of tumor immune invasion is complex, and not much is known. There have been reports that non-translocation-associated sarcomas have higher numbers of TILs than translocation-associated sarcomas, with DDLPS having the highest number of TILs among any other histological types [14], consistent with our results. There have been previous pan-cancer analyses that suggest that TIL burden is negatively correlated with copy number alterations [16]. DDLPS and PLPS are considered copy-number-driven sarcomas with low somatic mutation rates[1], highlighting the complex nature of mechanisms that drive infiltration of TILs. Furthermore, tumor associated macrophages (TAMs) are also an important component of the tumor immune microenvironment, and recent reports have demonstrated that the number of TAMs is significantly higher than that of TILs in many sarcomas, indicating a uniquely important role of macrophages in the tumor immune microenvironment of sarcomas [17]. It has been suggested that TAMs be classified into M1-like antitumoral macrophages and M2-like pro-tumoral macrophages [18], and M2-like macrophages are considered to have an important role in tumor progression [18, 19]. CD163 have been used as useful markers of M2-like macrophages, and higher infiltration of CD163+ macrophages is generally correlated with poor prognosis in several malignancies [19, 20]. For sarcomas, TAMs have been associated with poor prognosis in Ewing sarcomas [21] and synovial sarcomas [12]. Higher infiltration of CD163+ macrophages has also been correlated with poor prognosis in MLPS [22], consistent with our results. Interestingly, in our study, DDLPS patients with higher infiltration of CD163+ macrophages showed a trend toward favorable outcome, which was also seen in the results of Dancsok et al. [17], perhaps pointing to the uniqueness of DDLPS and complexity of how CD163+ macrophages contribute to disease progression. The CD47/signal-regulatory protein α (SIRP*α*) complex is key macrophage-related immune check point, which has been increasingly recognized as a promising therapeutic target in DDLPS [23, 24]. In LPS, the prognostic impact of CD47/SIRP*α* signaling in patients has not been reported, although many patients have been found to have CD47 expression in tumor cells, as well as infiltrating SIRPα positive macrophages [17]. Further studies uncovering the role of CD47/SIRP*α* signaling in LPS are warranted.

The recent success of immune checkpoint inhibitors, such as PD-1 or PD-L1 inhibitors in some malignancies, has garnered increased interest in immunomodulatory therapies [25, 26]. In the phase 2 clinical trial of anit-PD-1 inhibitor pembrolizumab in advanced soft tissue sarcomas (SARC028) [27], it was noted that higher baseline density of TILs in the tumor immune microenvironment was correlated with objective response rate [28], and patients with a B cell rich immune signature demonstrated high response rates [29]. Furthermore, although efficacy of pembrolizumab was limited in this study, objective response was achieved in two of ten patients with DDLPS [27]. While it is notable that the association between PD-L1 expression and response to immune check point inhibitors remains unclear, our results suggest that DDLPS and PLPS, which accumulate higher mutational burden than MLPS, provide higher immunogenicity, and are more likely to respond to immune checkpoint inhibitors than MLPS. The current understanding of the molecular biology of PLPS is limited, and efficacy of immune checkpoint inhibitors in PLPS remains unclear. However, our findings may indicate that anti-PD-1 therapy may be also promising in patients, considering the similar tumor immune microenvironment of PLPS to that of DDLPS.

Considering that MLPS are translocation-driven sarcomas with a low mutation burden and T cell infiltration, it seems that immunostimulatory approaches may be suitable for MLPS. Immunostimulatory therapies employing adoptive T cell transfer such as genetically engineered T cell receptor therapy and chimeric antigen receptor therapy have demonstrated dramatic effects in some malignancies [30, 31]. Immunostimulatory therapy has been of particularly high interest in sarcomas, since a majority of patients with MLPS express highly immunogenic cancer–testis antigen New York esophageal squamous cell carcinoma 1 (NY-ESO-1) [32, 33]. NY-ESO-1 is considered to be an attractive immunotherapeutic target because cancer–testis antigens are expressed only in germ cells of the testis but not in other adult tissues and are atypically re-expressed in various malignant tumors [32, 33]. NY-ESO-1 is also expressed in approximately 80% of synovial sarcoma [34] and immunotherapies with an autologous T cell transduced with a T cell receptor directed against NY-ESO-1 have demonstrated efficacy in patients with metastatic or refractory synovial sarcoma [35]. While immunotherapies against NY-ESO-1 are promising for patients with MLPS, antigen-specific adoptive T cell therapies require HLA class I expression on targeted cells for recognition. Antigen presentation by HLA class I expression on tumor surface is essential for the recognition of tumor cells by conventional CD8+ T cells. It is also known that loss or down regulation of HLA class I molecules is a common mechanism for tumor cells to escape from recognition by CD8+ T cells [36]. In the past, Pollack et al. [37] have suggested that MLPS may evade immune recognition through expression of a lower level of HLA class I; however, this has only been indicated by evaluating gene expression by RNA-seq. In our study, we report that protein expression of HLA class I is lost or downregulated in a majority of MLPS; furthermore, we found that all 16 metastatic specimens showed loss of HLA class I expression. Although further analysis to clarify the underlying mechanisms of downregulation of HLA class I in MLPS is warranted, these results suggest that the loss or downregulation of HLA class I expression may be a substantial obstacle in T cell-based immunotherapies for patients with MLPS. Zhang et al. [36] performed interferon-*γ* (IFN-*γ*) treatment in patients with synovial sarcoma and MLPS and demonstrated that IFN-*γ* treatment can increase expression level of HLA class I and PD-L1. Interestingly, this study included two patients with MLPS, and both patients were negative for HLA class I initially, but expression level of HLA class I became detectable after the IFN-*γ* treatments. Significantly this implies that the tumor immune microenvironment in patients with MLPS could be manipulated to facilitate immunotherapies including both immunomodulatory therapy and immunostimulants therapy. Considering the inhibitory role of HLA class I in NK cell function, our results also suggest NK cell therapies could be a promising treatment option for patients with MLPS [38]. Although the effect of adoptive transfer of NK cell therapy has been demonstrated in hematologic malignancies, the efficacy of NK cell therapy in solid tumors has been limited to early stage patients who have minimal residual tumor [39]. Our findings demonstrating the lack of HLA class I in MLPS suggests that in MLPS, adoptive NK cell therapies could be a promising treatment option for patients where wide resections are not possible or among those who have metastatic disease.

There are several limitations to be noted. First, despite the large number of MLPS patients, the number of DDLPS and PLPS patients enrolled in the current study is relatively low. These may have led to some inconclusive findings in the survival analyses for DDLPS and PLPS that did not reach statistical significance. Second, in many cases, molecular confirmation of the diagnosis was not available, and there is a possibility that there are patients subclassified inaccurately, which may impact the results. Third, while there are now many immune checkpoint markers known, we only evaluated PD-L1 expression. It is possible that MLPS tumors express other immune checkpoint molecules that we did not investigate, such as PD-L2 and TIM-3, among many [40]. Furthermore, although additional biomarkers such as tumor mutation burden, microsatellite instability, and DNA mismatch repair functional status have shown predictive response to immune checkpoint blockade, we did not investigate these biomarkers in this study [41–43].

Finally, a recent report from Petitprez et al. [29] has demonstrated that a strong B lineage gene signature determined by the MCP-counter tool was significantly associated with improved overall survival regardless of other immune factors such as high or low CD8+ TILs or FOXP3+ Tregs. Furthermore, a subclassification of patients treated on SARC028 demonstrated that a group defined by a unique immune profile characterized by the high density of B cells and presence of tertiary lymphoid structure could yield the highest response rate to PD-1 blockade therapy, further highlighting the importance of B cells in the tumor microenvironment in sarcomas. In our study, we did not evaluate for the infiltration of B cells, which clearly is a limitation. Further analysis will be necessary to evaluate the contribution of B cells in the LPS microenvironment. Additional larger scale studies are necessary to further dissect how various LPS tumors manipulate their immune microenvironment to evade immune surveillance and to assess the role of immunotherapeutic approaches in LPS.

In conclusion, here we have demonstrated that MLPS have a distinct tumor immune microenvironment from other LPS subtypes. While the overall number of infiltrating TILs and macrophages in MLPS patients were significantly less than in patients with DDLPS or PLPS, those with high macrophage numbers were shown to have poor outcome. In addition, loss or down regulation of HLA class I was frequently found in patients with MLPS. Furthermore, no patients with MLPS were positive for PD-L1, whereas about one quarter of patients with DDLPS and PLPS were positive. Overall, the tumor immune microenvironment of the translocation-associated MLPS is markedly different from the non-translocation-associated DDLPS and PLPS, suggesting that current approaches to cancer immunotherapies consisting of immunostimulatory and immunomodulatory approaches [25, 26, 35] may not be as effective in MLPS compared to other subtypes of LPS.

## Supplementary Information

Below is the link to the electronic supplementary material.

Supplementary file1 (PDF 52 kb)

Supplementary file2 (PDF 726 kb)
