# Supplementary material for: Human leukocyte antigen I is significantly downregulated in patients with myxoid liposarcomas
Source: Cancer Immunol Immunother. 2021 Apr 24;70(12):3489–99. doi: 10.1007/s00262-021-02928-1 (PMC8571150; doi:10.1007/s00262-021-02928-1)
Supplement: Supplementary file 1 — Supplementary file1 (PDF 52 kb) [file 262_2021_2928_MOESM1_ESM.pdf]

Supplementary table. 1

Marker	Clonality	Clone name	Species	Dilution	Vendor	Positive control	Negative control
CD4	Monoclonal	4B12	Mouse	1:50	Nichirei Bioscience, Tokyo, Japan	Tonsil	Tonsil (epithelial cells)
CD8	Monoclonal	C8/144B	Mouse	1:200	Nichirei Bioscience, Tokyo, Japan	Tonsil	Tonsil (epithelial cells)
FOXP3	Monoclonal	236A/E7	Mouse	1:200	Abcam, Cambridge, UK	Tonsil	Mouse IgG instead of first antibody
CD163	Monoclonal	10D6	Mouse	1:500	Leica, Wetzlar, Germany	Tonsil	Tonsil (epithelial cells)
HLA class I	Monoclonal	EMR8-5	Mouse	1:500	Hokudo, Sapporo, Japan	Internal control (endothelial cells)	Dermatofibrosarcoma protuberans
PD-L1	Monoclonal	28-8	Rabbit	1:200	Abcam, Cambridge, UK	Non-small cell lung cancer	MCF-7 (Brest cancer cell line)
