# Supplementary material for: Human leukocyte antigen I is significantly downregulated in patients with myxoid liposarcomas
Source: Cancer Immunol Immunother. 2021 Apr 24;70(12):3489–99. doi: 10.1007/s00262-021-02928-1 (PMC8571150; doi:10.1007/s00262-021-02928-1)
Supplement: Supplementary file 2 — Supplementary file2 (PDF 726 kb) [file 262_2021_2928_MOESM2_ESM.pdf]

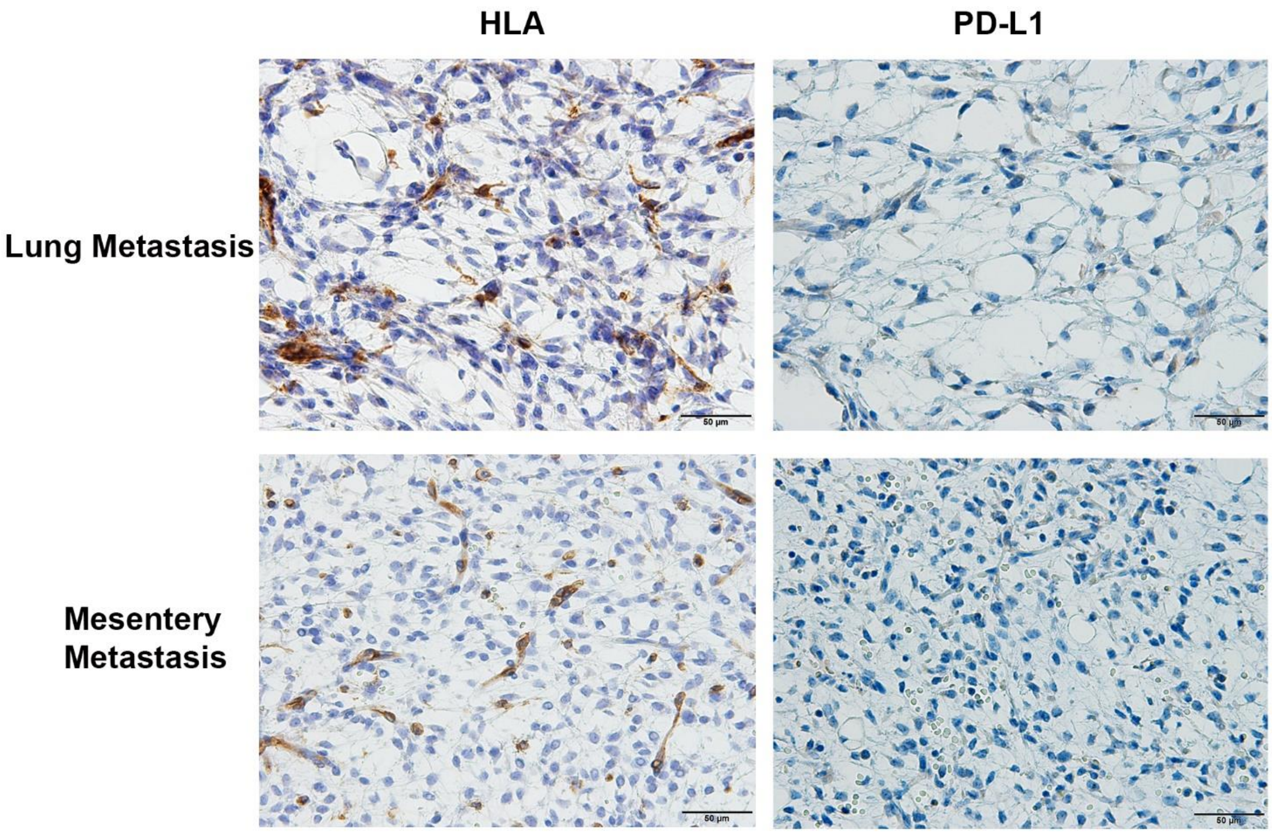

Supplementary Figure.1 Expression of HLA class I and Programmed death ligand 1 (PD-L1) in metastatic specimens of myxoid liposarcoma.

Both patients with lung metastases and mesentery metastasis were also negative for HLA class I (Left) and PD-L1 (Right). Scale bar represents 50 µm

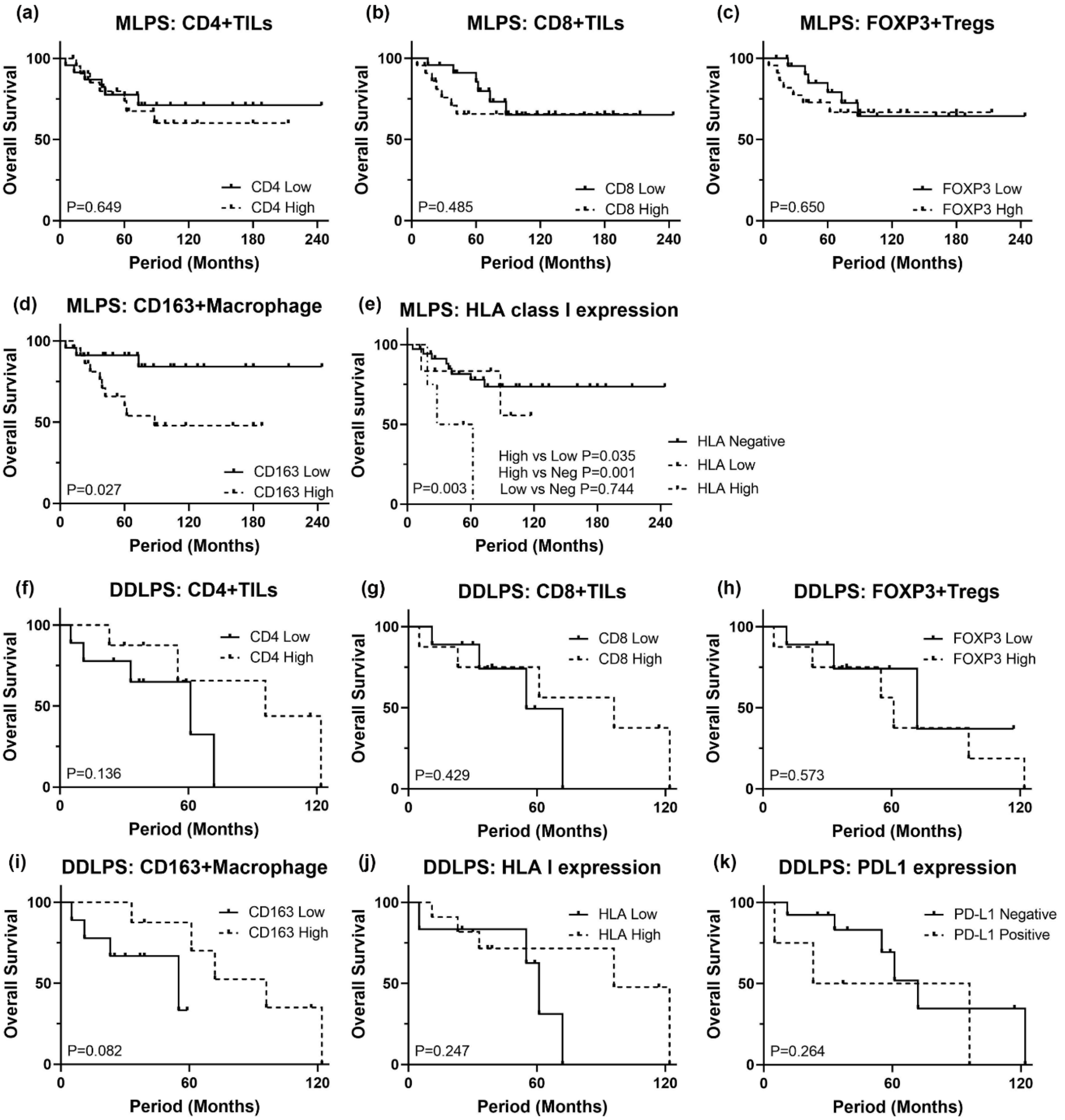

Supplementary Figure.2 Kaplan-Meier curves illustrating Overall survival (OS) in MLPS and DDLPS. The number of CD4+ TILs, CD8+ TILs, or FOXP3+Tregs do not show impact on OS in MLPS patients **(a-c)** while higher number of CD163+ macrophages or negative expression of HLA class I were associated with unfavorable OS in patients with MLPS **(d, e)**. Although no immune characteristics were found to be significantly associated with difference of OS in patients with DDLPS, patients with higher

infiltration of CD163 macrophages showed a trend toward favorable prognosis. **(f-k)**.

MLPS: Myxoid liposarcoma, DDLPS: Dedifferentiated liposarcoma
